# Transcriptome Analysis Reveals the Mechanism of Fluoride Treatment Affecting Biochemical Components in *Camellia sinensis*

**DOI:** 10.3390/ijms20020237

**Published:** 2019-01-09

**Authors:** Jiaojiao Zhu, Junting Pan, Shouhua Nong, Yuanchun Ma, Anqi Xing, Xujun Zhu, Bo Wen, Wanping Fang, Yuhua Wang

**Affiliations:** 1 College of Horticulture, Nanjing Agricultural University, Nanjing 210095, China; 2016104092@njau.edu.cn (J.Z.); panjunting@ibcas.ac.cn (J.P.); 2017804197@njau.edu.cn (S.N.); myc@njau.edu.cn (Y.M.); 2017104087@njau.edu.cn (A.X.); zhuxujun@njau.edu.cn (X.Z.); njauwb@njau.edu.cn (B.W.); fangwp@njau.edu.cn (W.F.); 2 Key Laboratory of Photobiology, Institute of Botany, Chinese Academy of Sciences, Beijing 100093, China

**Keywords:** *Camellia sinensis*, fluoride, RNA-Seq, transcriptome, bioactive compounds

## Abstract

Tea (*Camellia sinensis* (L.) O. Kuntze), one of the main crops in China, is high in various bioactive compounds including flavonoids, catechins, caffeine, theanine, and other amino acids. *C. sinensis* is also known as an accumulator of fluoride (F), and the bioactive compounds are affected by F, however, the mechanism remains unclear. Here, the effects of F treatment on the accumulation of F and major bioactive compounds and gene expression were investigated, revealing the molecular mechanisms affecting the accumulation of bioactive compounds by F treatment. The results showed that F accumulation in tea leaves gradually increased under exogenous F treatments. Similarly, the flavonoid content also increased in the F treatment. In contrast, the polyphenol content, free amino acids, and the total catechins decreased significantly. Special amino acids, such as sulfur-containing amino acids and proline, had the opposite trend of free amino acids. Caffeine was obviously induced by exogenous F, while the theanine content peaked after two day-treatment. These results suggest that the F accumulation and content of bioactive compounds were dramatically affected by F treatment. Furthermore, differentially expressed genes (DEGs) related to the metabolism of main bioactive compounds and amino acids, especially the pivotal regulatory genes of catechins, caffeine, and theanine biosynthesis pathways, were identified and analyzed using high-throughput Illumina RNA-Seq technology and qRT-PCR. The expression of pivotal regulatory genes is consistent with the changes of the main bioactive compounds in *C. sinensis* leaves, indicating a complicated molecular mechanism for the above findings. Overall, these data provide a reference for exploring the possible molecular mechanism of the accumulation of major bioactive components such as flavonoid, catechins, caffeine, theanine and other amino acids in tea leaves in response to fluoride treatment.

## 1. Introduction

Tea (*Camellia sinensis* (L) O. Kuntze) drinking is the main source of fluoride (F) in the human body [1]; owing to the high rate (40% to 90%) of soluble F in tea [2,3]. The ingestion of suitable amounts of F from tea is beneficial to human bones, tissues, and teeth [4]; but long-term drinking of certain types of tea (e.g., brick tea) increases the risk of diseases such as dental fluorosis and skeletal fluorosis [5,6]. Therefore, the influence of F on the quality of tea is related to human health. The tea plant (*Camellia sinensis*) is a hyperaccumulator of F, meaning the content of F is much higher than in other edible plants. Previously, Shu et al. (2003) reported that *C. sinensis* can accumulate 871–1337 mg·kg^−1^ F in mature leaves without showing any toxicity [7], which is 10–100 times higher than the levels of F in other plant species growing in the same environmental conditions [8]. The absorption of F in *C. sinensis* is very strong, especially in mature and old leaves of *C. sinensis*, where the enrichment rate of F could be as high as 98% of the total fluoride content of tea plants [9,10]. However, the mechanism for the hyperaccumulation of F in *C. sinensis* leaves remains unknown. 

The secondary metabolites in *C. sinensis*, especially polyphenols, caffeine, and amino acids, are essential to the flavor, taste, and nutrition of tea, which determine the quality of tea [11]. Previous studies have shown that the main bioactive components in *C. sinensis* can be affected by various environmental stress. For example, Wang et al. (2016) reported that drought stress adversely affects the quality of tea products by altering the accumulation of the main bioactive components, especially flavonoids, caffeine, theanine, and other amino acids [12]. Similarly, Li et al. (2018) preliminarily demonstrated that the accumulation of tea polyphenols in young leaves of *C. sinensis* increased in response to freezing stress, while amino acids decreased in response to freezing stress [13]. The accumulation of theanine in *C. sinensis* leaves was found to be reduced by sub-high temperatures via inhibiting the transcription of theanine biosynthesis genes [14]. In addition, Duan et al. (2012) found that the intensity of acid rain and the level of aluminum in the soil are closely related to the accumulation of major bioactive components in leaves of *C. sinensis* [15]. More interestingly, recent studies have shown that low concentration of F is beneficial to the synthesis of the main biologically active ingredients and the growth of *C. sinensis*, while F toxicity can reduce the content of the main biologically active ingredients and inhibit growth of *C. sinensis* [16,17]. For example, Li and Ni (2009) suggest that low concentrations of F increase the content of catechins by regulating the activity of catechins biosynthesis-related enzymes, while concentrations of F more than 8 mg·L^−1^ inhibit the growth of *C. sinensis* and the synthesis of catechins [18]. Therefore, the content of F in *C. sinensis* leaves is even employed as one of the indicators to evaluate the quality of tea [19]. On the other hand, Li et al. (2017) used RNA-Seq and digital gene expression (DGE) techniques to explore the accumulation mechanism of F in *C. sinensis*, and the results suggest that F uptake is associated with calcium transport ATPase, especially Ca^2+^ ATPase (ACAs) activated by RLK, as a carrier for the absorption of F in *C. sinensis* [20]. However, the molecular mechanism by which F affects the main bioactive components of tea leaves remains unclear.

In this study, the effects of F treatment on F accumulation and the main bioactive components, comprising polyphenols, flavonoids, and free amino acids, in tea leaves were investigated to reveal the molecular mechanism of F treatment. Furthermore, we also used HPLC and an amino acid automatic analyzer to detect changes in the accumulation of caffeine, individual catechins, theanine, and 17 hydrolyzed amino acids in tea leaves under F treatment. In addition, differentially expressed genes (DEGs) related to secondary metabolism and amino acid metabolism in *C. sinensis* leaves were identified for a clear understanding of the molecular mechanisms underlying the changes in the main bioactive components of tea leaves in the F treatment. The results indicate that F treatment further affects the accumulation of major bioactive components in tea leaves by modulating the transcription of genes related to the synthesis or metabolism of major bioactive components.

## 2. Results

### 2.1. The Changes in F, Polyphenol, Flavonoid, and Free Amino Acid Contents under F Treatment

**Figure 1:**
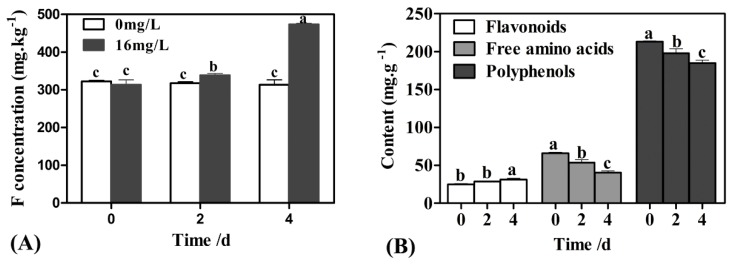
Effects of F treatment on the contents of fluoride (F), polyphenols, flavonoids and free amino acids in *Camellia sinensis* leaves. (**A**) The content of F in *C. sinensis* leaves significantly increased after treated with 16 mg/L F. (**B**) The contents of flavonoids, free amino acids and polyphenols in *C. sinesis* leaves were changed after treated with 16 mg/L F.

The results showed the accumulation of F in *C. sinensis* leaves treated with 16 mg/L F increased significantly with prolonged F exposure, especially after four days of treatment (Figure 1A). This suggests that the concentration of F in tea leaves was greatly affected by F in the culture medium. Additionally, the flavonoid, free amino acid, and polyphenol content in samples at different time points (zero, two, and four days) in F treatments were detected. As shown in Figure 1B, the flavonoids in the tea sample gradually increased after F treatment. Conversely, the accumulation of free amino acids and polyphenols was significantly decreased in response to F treatment at two and four days.

### 2.2. The Effect of F Treatment on Catechins and Caffeine Content

Individual catechins and caffeine in *C. sinensis* were determined by HPLC, and Table 1 shows the change of six individual catechins and the caffeine content in tea leaves in the 16 mg/L F treatment after zero, two, and four days of exposure. In general, F treatment significantly decreased total content of catechins compared to the control (zero days). The accumulation of gallocatechin (GC), epigallocatechin (EGC), epicatechin (EC), and epigallocatechin gallate (EGCG) in the tea samples treated with F was significantly reduced, while there existed no obvious change in catechin (C) and epicatechin gallate (ECG) contents. In addition, F treatment rapidly increased the accumulation of caffeine in *C. sinensis* leaves.

### 2.3. The Effect of the F Treatment on the Content of Theanine and Hydrolyzed Amino Acids

The results from the HPLC analysis used in the detection of theanine in tea leaves and the changes in its concentration are given in Table 2. The results showed that the accumulation of theanine in tea leaves increased significantly from 5.817 mg·g^−1^ (zero days) to 6.655 mg·g^−1^ (two days) and then decreased to 6.164 mg·g^−1^ (four days). In addition, 17 hydrolyzed amino acids were determined by an automatic amino acid analyzer. The content of 13 amino acids, Thr, Ser, Asp, Glu, Gly, Ala, Val, Ile, Leu, Phe, His, Arg, and Lys, in response to F was lower relative to the control group (zero days). Particularly, the His, Arg, Asp, and Glu were significantly lower than that of the control. Conversely, the content of four amino acids, Cys, Met, Tyr, and Pro, increased gradually in response to F treatment; with a significant increase in Cys content. These results therefore, suggest that F treatment does not favor synthesis of total amino acids in tea leaves.

### 2.4. Identification and Analysis of DEGs Associated with Amino Acid Metabolism and Secondary Metabolism

**Figure 2:**
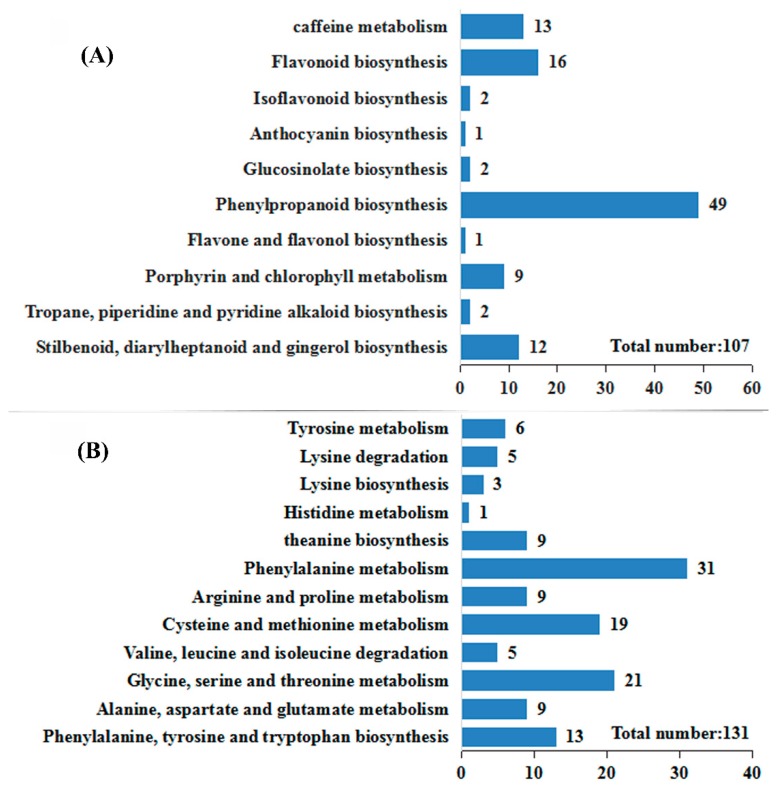
KEGG ontology (KO) enrichment analysis of DEGs related to secondary metabolism and amino acid metabolism in *C. sinensis* leaves under F treatment. A total of 107 and 131 DEGs were annotated and found to be associated with secondary metabolism (**A**) and amino acid metabolism (**B**), respectively, based on the KEGG database.

According to the transcriptome data (NCBI SRA Accession Number PRJNA512448), 107 and 131 DEGs were found to be associated with secondary metabolic pathways and amino acid metabolism, respectively (Tables S1 and S2). The result presented in Figure 2A shows that the highest proportion of DEGs was associated with the phenylpropanoid biosynthetic pathway in all secondary metabolic pathway-related DEGs. F treatment mainly affects the secondary metabolism of tea leaves by changing phenylpropanoid metabolism. Interestingly, as shown in Figure 2B, 31 DEGs were associated with the phenylalanine metabolism pathway in the amino acid metabolism pathway, suggesting that phenylalanine metabolism plays an important role in the amino acid metabolism pathway in *C. sinensis* leaves.

### 2.5. Changes in the Flavonoid, Caffeine, and Theanine Biosynthesis Pathways in C. sinensis in Response to F Treatment

**Figure 3:**
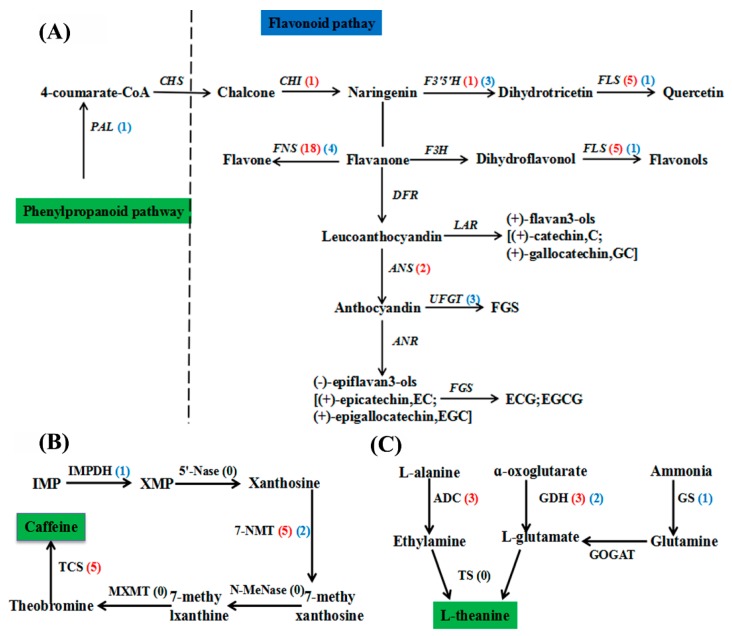
Differentially expressed genes (DEGs) involved in flavonoid, caffeine and theanine biosynthesis in *C. sinensis* leaves under F treatment. (**A**) The flavonoid biosynthesis pathway. PAL, phenylalanine ammonia lyase; CHI, chalcone isomerase; F3′5′H, flavonoid 3′,5′-hydroxylase; FLS, flavonol synthase; FNS, flavone synthase; F3H, flavanone 3-hydroxylase; DFR, dihydroflavonol 4-reductase; LAR, leucoanthocyanidin reductase; ANS, anthocyanidin synthase; ANR, anthocyanidin reductase; FGS, flavan-3-ol gallate synthase; UFGT, UDP-glucose: flavonoid 3-O-glucosyl transferase. (**B**) The caffeine biosynthesis pathway. IMPDH, IMP dehydrogenase; 5′-Nase, 5′-nucleotidase; 7-NMT, 7-methylxanthosine synthase; N-MeNase, N-methylnucleotidase; MXMT, theobromine synthase; TCS, tea caffeine synthase. (**C**) The theanine biosynthesis pathway. ADC, arginine decarboxylase; TS, theanine synthetase; GS, glutamine synthetase; GDH, glutamatedehydrogenase; GOGAT, glutamate synthase. The numbers in parentheses following each gene name indicate the number of corresponding DEGs, red indicates that the differentially expressed genes are up-regulated and blue is down-regulated.

Thirty-nine DEGs were identified from the transcriptome data to participate in the regulation of flavonoids biosynthesis pathway (Table S3). All known genes associated with the flavonoid biosynthesis pathway in *C. sinensis* treated with F, such as phenylalanine ammonia lyase (PAL, 1 unigene), chalcone isomerase (CHI, 1 unigene), flavonoid 3′,5′-hydroxylase (F3′5′H, 4 unigenes), flavonol synthase (FLS, 6 unigenes), flavone synthase (FNS, 22 unigenes), anthocyanidin synthase (ANS, 2 unigenes), UDP-glucose: Flavonoid 3-*O*-glucosyl transferase (UFGT, 3 unigenes), are shown in Figure 3A. In addition, unigenes related to flavonoid synthesis, such as FNS and FLS, were up-regulated after two days of F treatment, which may be the reason for the increase in total flavonoids content under F treatment (Figure 3A and Figure 1B). Chalcone was synthesized by 4-coumarate CoA under the action of chalcone synthase, and the down-regulation of a PAL-related unigene in the flavonoid synthesis pathway in the F treatment leading to a decrease in chalcone content. Since the synthesis of flavonoids and catechins are closely related to chalcone, the increase in flavonoid content will inevitably lead to a decrease in the content of total catechins, which is consistent with the change in the content of catechins identified in the HPLC analysis.

In the transcriptome analysis, 13 DEGs were associated with the caffeine biosynthesis, including 7-methylxanthosine synthase (7-NMT, 7 unigenes), IMP dehydrogenase (IMPDH, 1 unigenes), and tea caffeine synthase (TCS, 5 unigenes; Figure 3B; Table S4). The changes of DEGs related to caffeine synthesis in the F treatment are shown in Figure 2B. Specifically, most of the DEGs associated with caffeine synthesis in tea leaves were up-regulated, which also explained the increase of caffeine content in tea leaves after two days of F treatment.

As shown in Figure 3C, nine genes related to theanine biosynthesis, such as glutamate dehydrogenase (GDH, 5 unigenes), arginine decarboxylase (ADC, 3 unigenes), and glutamine synthetase (GS, 1 unigenes), were differentially expressed in tea leaves treated with F for two days, relative to the control (Table S5). Except for two GDH unigenes and one GS unigene, all of the unigenes were up-regulated in response to F treatment (Figure 3C), which is consistent with the investigated increase in theanine accumulation in response to two days of F treatment (Table 2).

### 2.6. qRT-PCR Validation of DEGs from RNA-seq

**Figure 4:**
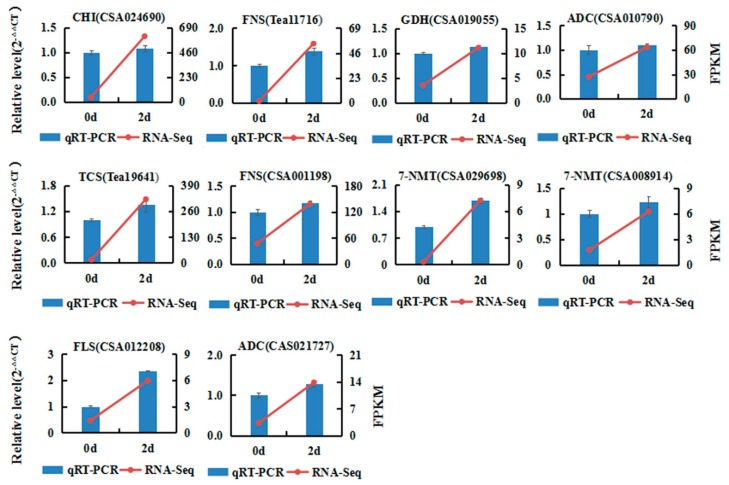
Verification of the relative expression levels of DEGs by qRT-PCR. Expression patterns of 10 DEGs related to the theanine, caffeine, and flavonoid biosynthetic pathways by qRT-PCR (blue bar) and RNA-Seq (red line).

Among the 10 DEGs selected for qRT-PCR analysis, four, three, and three DEGs were associated with flavonoids, caffeine, and theanine biosynthesis pathways, respectively. These were then used to verify the correctness of the expression profile of unigenes obtained by the Illumina RNA-Seq analysis. Ten primers, namely FNS (Tea11716 and CSA001198), CHI (CSA024690), FLS (CSA012208), TCS (Tea19641), 7-NMT (CSA008914 and CSA029698), GDH (CSA019055), and ADC (CSA010790 and CSA021727) were designed from the identified DEGs for gene expression analysis, as shown in Table S6. The results of the qRT-PCR were consistent with the estimated transcript results from the RNA-Seq output (Figure 4), suggesting the reliability of the RNA-Seq results.

## 3. Discussion

Fluoride is not an essential element for plants and may directly or indirectly inhibit the physiological and biochemical processes by disrupting metabolic processes, such as respiration and photosynthesis [21], ultimately leading to slow growth and plant death [22]. Similarly, increasing research indicates that F is not an essential element of tea plants [23], and high concentrations of F not only have a significant effect on the accumulation of F in *C. sinensis*, but also contribute to the accumulation of major bioactive components in tea plants [24]. Therefore, the content and proportion of bioactive components in tea leaves directly determine the quality and value of the tea [25]. Consistent with a report by Li et al. (2017) [20], the results in the presents study showed that F content in leaves of *C. sinensis* increased significantly with F treatment over time.

The polyphenol and amino acid content in *C. sinensis* are closely related to the quality of the tea [26]. Specifically, polyphenols are essential bioactive components in tea leaves [27], and the change of polyphenol content in *C. sinensis* leaves influences tea quality [28,29], while amino acids are the main constituents of the freshness and mellowness of tea [30]. The effect of F treatment on the accumulation of tea polyphenols and amino acids in tea leaves was also investigated in this study. The results revealed a significant reduction in polyphenols and amino acids content in tea leaves, with F treatment. It can therefore be speculated that this change is not conducive to the formation of high quality tea. This result is consistent with reports by Li et al. (2009) [18].

As a component of polyphenols, flavonoids protect plant tissues from the stresses of various external environments, and the biosynthesis of flavonoids increases in response to environmental stress such as drought stress [31], low temperature stress [32], excessive ultraviolet light [33], and pathogen infection [34]. Simultaneously, catechin is the main component of tea polyphenols, which accounts for approximately 70% of total tea polyphenols [35]. Catechin is synthesized by secondary metabolic pathways such as the shikimate pathway, the phenylpropanoid pathway, and the flavonoid pathway. Catechins are closely related to the taste and quality of tea. Research has shown that tea leaves and buds are rich in catechins, including esterified catechins and non-esterified catechins [36]. An increasing number of studies have shown that environmental stress, such as drought [37,38], low temperature [39], and fluoride [16], affect the accumulation of catechins in tea leaves. In the present study, we not only investigated the effects of treatment with 16 mg/L F on the accumulation of flavonoids and total catechins in tea leaves, but also identified the DEGs related to the biosynthesis pathway of flavonoids and total catechins by RNA-Seq. The results revealed that the accumulation of flavonoids in tea leaves increased with prolonged F treatment, implying that flavonoids could respond to exogenous F, however, F treatment also inhibited the accumulation of total catechins in tea leaves, which is consistent with the results described by Li et al. (2015) [16]. Furthermore, the transcriptome analysis showed that most of the known genes associated with flavonoid biosynthesis were up-regulated in the F treatment. In particular, the up-regulated expression of DEGs associated with flavone synthase (FNS), flavonol synthase (FLS), chalcone isomerase (CHI), and anthocyanidin synthase (ANS) might explain the increase in flavonoid content in tea leaves under F treatment (Figure 1B). In addition, phenylalanine ammonia lyase (PAL) is a key rate-limiting enzyme that links primary metabolism and phenylpropanoid metabolism. It is also the starting site of the metabolic pathway, and the activity of the enzyme directly determines the level of bioactive components in the subsequent series of sub-biomass synthesis steps. We hypothesized that the PAL gene that is down-regulated in the flavonoid synthesis pathway in the F treatment can explain the decrease in the content of total catechins. In summary, these results suggest that the changes of catechins and flavonoids in tea leaves in the F treatment depend on the regulation of genes involved in the biosynthesis of flavonoids.

Caffeine is a purine alkaloid and a major bioactive compound in *C. sinensis* [11]. Caffeine is mainly synthesized via a typical caffeine biosynthesis pathway in the young leaves of *C. sinensis* [40]. This study analyzed the DEGs associated with the caffeine synthesis pathway, and the results showed that the DEGs associated with the caffeine synthesis pathway, 7-NMT and TCS, were mostly up-regulated, which confirmed the observed increase of the caffeine content in *C. sinensis* treated with F. Overall, F treatment increases caffeine accumulation in tea leaves by promoting the expression of genes associated with caffeine biosynthesis, such as 7-NMT and TCS.

Theanine is a natural amino acid unique to *C. sinensis* that is closely related to tea quality [14]. Similar to other biochemical components, the content of theanine in *C. sinensis* is also affected by environmental stress. For example, Li et al. (2018) concluded that sub-high temperature treatment reduced the synthesis of theanine in tea leaves by inhibiting the expression of genes involved in the synthesis of theanine biosynthesis. Deng et al. (2012) fully demonstrated that salt stress affects the accumulation of theanine in roots and shoots by altering the synthesis of theanine synthase [41]. The conclusions of this study indicate that the biosynthesis of theanine in tea leaves is derived from glutamate and ethylamine and is regulated by downstream GS, GOGAT, GDH, ADC, and TS (Figure 3C). Combining the results of the RNA-Seq and HPLC analyses, we believe that the significantly increased theanine content in response to two days of F treatment is due to the up-regulation of DEGs associated with the synthesis of theanine. However, the decrease in the content of theanine after four days of F treatment can explain the long-term exposure of *C. sinensis* to high concentrations of F, which is not conducive to the synthesis of theanine. Based on the above analyses, we hypothesized that the change in the content of theanine under F treatment mainly depends on the change of the differentially expressed genes related to theanine synthesis. Furthermore, the reduced total hydrolyzed amino acid content in the F treatment is very unfavorable for the formation of high-quality tea. In addition, we identified DEGs associated with amino acid synthesis, suggesting a potential regulatory mechanism behind the above changes in amino acid content.

## 4. Materials and Methods

### 4.1. F treatment and Leaf Sampling

One-year-old cutting seedlings with consistent growth of *C. sinensis* cultivar ‘*Longjingchangye*’ was transplanted from the Nanjing Ya Run Tea Co., Ltd., Nanjing, China. The soil on the roots was washed with dd H_2_O, and then the seedlings were cultured in light incubators with photoperiod of 12-h light (25 °C ± 2 °C) /12-h dark (22 °C), The tea seedlings were first cultured in dd H_2_O for 3 days, and then cultured for 1 week with 1/2 complete nutrient solution, and finally cultured in the complete culture solution for 2 weeks. After that, the tea seedlings were divided into two groups; one group was incubated in the complete medium as control, the other group was incubated in the complete medium containing 16 mg/L F as treatment. The complete culture solution contains (in mM) (NH_4_)_2_SO_4_, 0.713; NH_4_NO_3_, 0.73: KH_2_PO_4_, 0.1; K_2_SO_4_, 0.46; CaCl_2_, 0.5; MgSO_4_, 0.41; Fe-EDTA, 0.032; H_3_BO_3_, 0.046; CuSO_4_, 0.002; MnSO_4_, 0.09; Na_2_MoO_4_, 0.0026; ZnSO_4_, 0.0091 according to the report by Ghanati et al. (2005) [42]. The nutrient solution was refreshed once a week, and the pH of the solution was maintained at 4.8–5.0 and adjusted by 1 M HCl or 1 M NaOH every day.

The young tea leaves (the first and the second leaves) were collected at 0 and 2 d and immediately frozen in liquid nitrogen and stored at −80 °C for RNA-seq and qRT-PCR assays. To detect the accumulation of F, the first and second leaves from about 30 tea seedlings treated with 16 mg/L F for 0, 2, and 4 days were collected and washed, then dried at 80 °C and ground into a fine powder. To detect the main bioactive components, the first and second leaves from 75 seedlings treated with 16 mg/L F for 0, 2, and 4 days were collected and washed, then dried at 80 °C and ground into a fine powder. All the extractions and measurements were repeated three times.

### 4.2. Extraction and Determination of F in Tea Samples

To investigate the F accumulation, 0.5 g finely powdered tea leaves was used to extract F. Then the F concentration was detected by fluoride ion selective electrode (Thermo Scientific Orion Star A214) according to the method proposed by Gao et al. (2012) [43].

### 4.3. Extraction and Determination of Polyphenol, Flavonoid, and Amino Acid

The polyphenols in 0.2 g powdered leaf samples were extracted with 70% methanol at 70 °C, and the content was evaluated using a UV-Visible spectrophotometer (MAPADA, Shanghai, China) at 765 nm according to the Folin–Ciocalteu method described by Li et al. (2015) [44]. The free amino acids in the *C. sinensis* samples were extracted in a boiling water bath for 45 min, and then determined according to the ninhydrin coloring method [45]. Determination of flavonoids content was carried out by AlCl_3_ method after extraction with deionized water at 100 °C [46]. The other amino acids in tea samples were extracted according to the method described by Wan et al. (2015) [47], and the contents were determined by using an L-8900 automatic amino acid analyzer (Hitachi, Japan).

### 4.4. Extraction and Determination of Theanine, Catechins, and Caffeine

Theanine was extracted from tea leaves with deionized water at 100 °C for 30 min, using 1 g fine powder, and then the extraction was filtered through a 0.45 µm Millipore filter prior to HPLC analysis. The theanine content was determined using a Shimadzu LC-20A HPLC system (Shimadzu, Japan) according to the method reported by Tai et al. (2015) [48]. The catechins and caffeine in the samples were extracted and determined according to the method recommended by Chen et al. (2015) using HPLC [49].

### 4.5. Construction of RNA Library and RNA Sequencing

Extraction of total RNA and RNA-seq were performed in three biological replicates. A total of 10 young leaves from five seedlings for each replicate of one sample were collected for extraction of RNA by RNAiso Plus (TaKaRa, Japan) following the manufacturer’s instructions. The quality and integrity of the RNA was determined by a NanoDrop ND-1000 spectrophotometer (NanoDrop, Wilmington, DE, USA) and a 2100 Bioanalyzer RNA Nano chip device (Agilent Technologies, Palo Alto, CA, USA). The same amount of RNA was then collected from the three biological replicates for cDNA preparation. cDNA was synthesized and sequenced using an Illumina HiSeq^TM^ 2000, and the results were analyzed according to the method described by Ren et al. (2014) [50]. Briefly, clean sequences were acquired by discarding adaptor sequences, and then the clean reads were then assembled into unigenes using the Trinity software [51]. Finally, BLASTX alignment (e <0.00001) was performed between unigenes and protein databases including KEGG, Swiss-Prot, NR, and COG, and the best aligning results were used to decide the sequence direction of the unigenes.

### 4.6. Identification and Analysis of DEGs Related to Amino Acid Metabolism and Secondary Metabolism

Differentially expressed genes (DEGs) were identified according to stringent criteria: The false discovery rate (FDR) <0.05 and the |log_2_Ratio| ≥2, and the expression of unigenes was calculated using the FPKM method. Based on the *C. sinensis* transcriptome database in the F treatment, and according to the results from the screening of DEGs and KEGG enrichment analysis, we obtained the amino acid metabolism of DEGs and secondary metabolites of DEGs. To analyze the molecular mechanism behind the accumulation of the main bioactive components in the F treatment, we further explored the DEGs associated with the synthesis of catechins, caffeine, and theanine in tea leaves.

### 4.7. Quantitative Real-Time PCR (qRT-PCR) Analysis of the Selected DEGs

In order to confirm the reliability of RNA sequencing, 10 DEGs were randomly selected for quantitative RT-PCR (qRT-PCR). The Csβ-actin gene (GenBank: HQ420251.1) of *C. sinensis* was used as the internal reference gene, and the relative expression levels were calculated by the 2^−ΔΔCt^ method [52]. The primers for qRT-PCR are shown in Supplementary S1.

### 4.8. Statistical Analyses

The statistical analysis of this paper is carried out according to the method of Wang et al. (2016) [53]. All of the tests in this study were performed in three biological replicates, with three separate extractions in each replicate. All the data of the experimental results are presented as the means ± standard deviations (SD). In order to detect differences between groups, a one-way ANOVA and Duncan’s test was performed, and different letters indicate the difference at *p* < 0.05 level. SPSS 22 software was used to analyze the data.

## 5. Conclusions

In conclusion, we found that F treatment adversely affects the accumulation of F and the main bioactive components (polyphenols, flavonoids, caffeine, theanine, and amino acids) of tea, indicating F is an important factor that reduces tea quality. The biosynthesis pathways of catechins (such as FNS, FLS, CHI, FLS, ANS, F3′5′H, and UFGT), caffeine (TCS and 7-NMT), and theanine (ADC and GDH) further confirmed the changes in the main bioactive compounds in leaves of *C. sinensis* in response to F treatment. Our results therefore reveal that F treatment alters the contents of the main bioactive components via affecting the gene expressions in their biosynthesis pathways, and ultimately reduces tea quality.

## Figures and Tables

**Table 1:** Changes in the amount of individual catechins and caffeine (mg·g^−1^) induced by F treatment.

Treatment Time (day)	GC	EGC	C	EC	EGCG	ECG	Caffeine
**0**	21.01 ± 0.01a	9.75 ± 0.40a	3.65 ± 0.09a	15.81 ± 0.16a	12.40 ± 0.19a	30.22 ± 0.60b	9.39 ± 0.07c
**2**	17.06 ± 0.07b	7.98 ± 0.13b	3.45 ± 0.06b	15.42 ± 0.25b	8.89 ± 0.07b	31.11 ± 010a	12.18 ± 0.02b
**4**	15.91 ± 0.24c	6.36 ± 0.38c	3.54 ± 0.12ab	14.09 ± 0.04c	5.12 ± 0.08c	29.53 ± 0.23c	12.35 ± 0.04a

**Table 2:** F treat-induced changes in content of theanine and other free amino acids (mg·g^−1^).

Amino Acid	Treatment Times (day)		
	0	2	4
The	5.817 ± 0.228c	6.655 ± 0.207a	6.164 ± 0.069b
Asp	1.369 ± 0.006a	1.341 ± 0.013b	1.327 ± 0.007c
Thr	0.665 ± 0.003a	0.661 ± 0.005a	0.651 ± 0.008b
Ser	0.703 ± 0.007a	0.690 ± 0.003ab	0.678 ± 0.008b
Glu	1.917 ± 0.009a	1.873 ± 0.005b	1.826 ± 0.018c
Gly	0.775 ± 0.003a	0.774 ± 0.008a	0.760 ± 0.009b
Ala	0.778 ± 0.003a	0.761 ± 0.006b	0.750 ± 0.010b
Cys	0.112 ± 0.001c	0.119 ± 0.002b	0.124 ± 0.002a
Val	0.773 ± 0.003a	0.762 ± 0.007ab	0.747 ± 0.011b
Met	0.153 ± 0.006a	0.162 ± 0.003a	0.165 ± 0.004a
Ile	0.592 ± 0.004a	0.584 ± 0.009a	0.577 ± 0.007a
Leu	1.192 ± 0.002a	1.177 ± 0.010ab	1.152 ± 0.020b
Tyr	0.425 ± 0.022b	0.451 ± 0.008ab	0.461 ± 0.008a
Phe	0.887 ± 0.010a	0.869 ± 0.003ab	0.851 ± 0.015b
Lys	1.078 ± 0.008a	1.060 ± 0.009ab	1.048 ± 0.014b
His	0.335 ± 0.001a	0.324 ± 0.006b	0.315 ± 0.002c
Arg	1.233 ± 0.043a	1.161 ± 0.008b	0.992 ± 0.007c
Pro	0.571 ± 0.007b	0.588 ± 0.010a	0.597 ± 0.011a

## Supplementary Materials

Supplementary materials can be found at http://www.mdpi.com/1422-0067/20/2/237/s1.

## Abbreviations

DEG	differentially expressed gene
HPLC	high performance liquid chromatography
qRT-PCR	quantitative real time PCR
RNA-Seq	RNA Sequencing
PAL	phenylalanine ammonia lyase
CHI	chalcone isomerase
F3′5′H	flavonoid 3′,5′-hydroxylase
FLS	flavonol synthase
FNS	flavone synthase
ANS	anthocyanidin synthase
UFGT	flavonoid 3-*O*-glucosyl transferase
7-NMT	7-methylxanthosine synthase
IMPDH	IMP dehydrogenase
TCS	tea caffeine synthase
GDH	glutamate dehydrogenase
ADC	arginine decarboxylase
GS	glutamine synthetase
NR	non-redundant
Swiss-Prot	swiss-prot database
KEGG	Kyoto Encyclopedia of Genes and Genomes
COG	Cluster of Orthologous Groups of proteins
F	fluoride
ACAs	Ca^2+^ ATPase
GC	gallocatechin
EGC	epigallocatechin
EC	epicatechin
EGCG	epigallocatechin gallate
C	catechin
ECG	epicatechin gallate
